# Surgical Complexity and Postoperative Outcomes in Recurrent Versus Primary Incisional Hernias Following Elective Retromuscular Repair: A Retrospective Cohort Study

**DOI:** 10.3390/diagnostics16162486

**Published:** 2026-08-07

**Authors:** Laurențiu Augustus Barbu, Daniel Ioan Mihalache, Nicolae-Dragoș Mărgăritescu, Liviu Vasile, Mirela Coroescu, Ioan Sorin Tudorache, Tiberiu Stefăniță Țenea Cojan, Marius P. Iordache, Anca Buliman

**Affiliations:** 1 Department of Surgery, Railway Clinical Hospital Craiova, University of Medicine and Pharmacy of Craiova, 2 Petru Rares Street, 200349 Craiova, Romania; laurentiu.barbu@umfcv.ro (L.A.B.); tiberiu.tenea@umfcv.ro (T.S.Ț.C.); 2 Doctoral School, “Carol Davila” University of Medicine and Pharmacy, 37 Dionisie Lupu Street, 020021 Bucharest, Romania; dan64mm@yahoo.com; 3 Department of Surgery, Emergency County Hospital, University of Medicine and Pharmacy of Craiova, 2 Petru Rares Street, 200349 Craiova, Romania; dmargaritescu@yahoo.com (N.-D.M.); vliviu777@yahoo.com (L.V.); 4 Department of Radiology, Faculty of Medicine, Titu Maiorescu University, 67A Gheorghe Petrașcu Street, 031593 Bucharest, Romania; coroescumirela@yahoo.com (M.C.); buliman_anca@yahoo.com (A.B.); 5 Department of Anatomy, Faculty of Medicine, Titu Maiorescu University, 67A Gheorghe Petrașcu Street, 031593 Bucharest, Romania; sorin.tudorache@prof.utm.ro; 6 Department of Clinical Neurology, Faculty of Medicine, Titu Maiorescu University, 67A Gheorghe Petrașcu Street, 031593 Bucharest, Romania

**Keywords:** incisional hernia, recurrent hernia, operative burden, surgical complexity, postoperative complications, abdominal wall reconstruction, Rives–Stoppa repair, risk stratification, surgical outcomes

## Abstract

**Background/Objectives:** Recurrent incisional hernia remains a major challenge in abdominal wall surgery and is frequently associated with increased surgical complexity and adverse outcomes. However, its role as a distinct surgical entity and its cumulative operative impact remain insufficiently characterized. **Methods:** We conducted a retrospective cohort study including 1262 patients undergoing incisional hernia repair using the Rives–Stoppa technique between 2013 and 2022. Patients were stratified into primary and recurrent hernia groups. Operative burden was assessed using operative time and length of hospital stay. **Results:** Recurrent hernia was associated with a more adverse clinical profile, increased structural complexity, longer operative time, prolonged hospitalization, and higher postoperative complication rates. It remained independently associated with postoperative complications after adjustment for the principal measured confounders. **Conclusions:** Recurrent incisional hernia was associated with increased structural complexity, longer operative time, prolonged hospitalization, and worse postoperative outcomes compared with primary incisional hernia in patients undergoing elective retromuscular repair. Moreover, recurrent hernia remained independently associated with postoperative complications after adjustment for the principal measured confounders. These findings should be interpreted within the context of elective Rives–Stoppa repair and require external validation before broader clinical application.

## 1. Introduction

Incisional hernia is one of the most common long-term complications following abdominal surgery and remains a major source of morbidity, impaired quality of life, and healthcare utilization [1]. Its incidence ranges from low percentages to more than 30% in high-risk populations, depending on patient- and procedure-related factors [2]. Beyond mechanical failure of the abdominal wall, incisional hernia is increasingly recognized as a multifactorial disease involving alterations in tissue remodeling and collagen metabolism that contribute to impaired wound healing and recurrence [3,4].

Despite advances in mesh-based abdominal wall reconstruction, recurrence remains a major clinical challenge. Recurrent incisional hernias are associated with altered anatomy, fibrosis, previous mesh implantation, and compromised tissue quality, making repair technically more demanding and increasing the risk of postoperative complications and healthcare resource utilization [5,6,7].

Current risk assessment is largely based on individual patient characteristics, such as obesity, diabetes, and defect complexity, or on preoperative scoring systems, including the Ventral Hernia Working Group classification and the CeDAR score [8,9,10,11,12,13,14]. Although these approaches identify important risk factors, they do not fully capture the cumulative impact of structural complexity, intraoperative demands, and postoperative resource utilization.

The present study aimed to evaluate the impact of recurrent incisional hernia on operative burden and postoperative outcomes in a large cohort of patients undergoing elective retromuscular repair.

## 2. Materials and Methods

### 2.1. Study Design and Population

A retrospective cohort study was conducted, including patients who underwent surgical repair of an incisional hernia using the retromuscular Rives–Stoppa technique. The study population consisted of 1262 consecutive patients treated at the Emergency County Hospital of Ploiești, Romania, between January 2013 and December 2022. Cases were identified through a retrospective review of the institutional electronic medical record system and operative databases. All consecutive eligible patients meeting the predefined inclusion and exclusion criteria during the study period were included. No sampling strategy was applied. Patients were stratified according to hernia status into primary and recurrent incisional hernia groups.

Primary incisional hernia was defined as an incisional hernia occurring in a patient without a previous history of incisional hernia repair. Owing to the retrospective nature of the study, detailed information regarding the index abdominal operation (elective versus emergency procedure, open abdomen management, or delayed fascial closure) was not consistently available and was therefore not included in the analysis.

#### Inclusion and Exclusion Criteria

Adult patients (≥18 years) undergoing elective incisional hernia repair were eligible for inclusion. Emergency incisional hernia repairs performed for acute incarceration, strangulation, bowel obstruction, perforation, or other urgent hernia-related complications requiring immediate surgery, patients with incomplete key clinical data, and cases treated using non-standard surgical techniques were excluded from the analysis.

### 2.2. Surgical Technique

All patients underwent open retromuscular mesh repair based on the Rives–Stoppa principle. For midline incisional hernias, a classic retromuscular sublay repair was performed with bilateral dissection of the retro-rectus space and placement of a prosthetic mesh in the retromuscular position. For lateral incisional hernias, the same retromuscular principle was applied whenever anatomically feasible, with mesh placement in the corresponding extraperitoneal plane and fixation adapted to the defect location. The fundamental concept of wide mesh overlap in a well-vascularized retromuscular/extraperitoneal space was maintained in both midline and lateral defects. Because of the retrospective design and the long study period, detailed information regarding mesh characteristics, fixation techniques, drain use, component separation procedures, closure adjuncts, and perioperative management protocols was not consistently available and could not be reliably analyzed. Nevertheless, the use of a standardized open retromuscular repair strategy based on the Rives–Stoppa principle provided a relatively homogeneous surgical framework for the present analysis.

### 2.3. Data Collection and Variables

Clinical, demographic, and operative data were extracted from institutional electronic medical records. All data were obtained from the electronic medical record system of the Emergency County Hospital of Ploiești. Investigators affiliated with the study center had authorized access to anonymized data in accordance with institutional policies and ethics committee approval. Baseline variables included age, body mass index (BMI), comorbidities (diabetes mellitus), smoking status, and American Society of Anesthesiologists (ASA) classification.

Hernia-related variables included defect size, categorized according to width (W2: 4–10 cm; W3: >10 cm), and anatomical location (midline vs. lateral).

Operative parameters included operative time and length of hospital stay (LOS). Surrogate markers of technical difficulty were defined a priori and included prolonged operative time (>150 min), presence of large defects (W3), and a composite high-risk profile (≥2 of the following: obesity, diabetes, ASA III–IV, W3 defect).

Postoperative complications were recorded during the index hospitalization and were defined as any documented deviation from the expected postoperative course requiring additional evaluation, treatment, or monitoring. Owing to the retrospective nature of the study, complications were analyzed as a composite outcome and were not systematically classified according to the Clavien–Dindo grading system. Outcomes included overall complications, prolonged hospitalization (>8 days), and need for reintervention.

### 2.4. Statistical Analysis

Data preprocessing and management were performed using Microsoft Excel 2016 (Microsoft Corp., Redmond, WA, USA) and XLSTAT 2022.5 (Addinsoft, Paris, France). Statistical analyses were conducted using IBM SPSS Statistics version 27.0 (IBM Corp., Armonk, NY, USA).

Categorical variables were expressed as frequencies and percentages and compared using the chi-square test. Continuous variables were reported as mean ± standard deviation and compared using Student’s *t*-test.

Multivariable logistic regression analyses were performed to identify variables independently associated with recurrent hernia and postoperative complications. Results were expressed as odds ratios (ORs) with 95% confidence intervals (CIs). In addition to *p*-values, absolute differences between groups and effect estimates with 95% confidence intervals were reported where appropriate to facilitate interpretation of the magnitude of observed associations. The analyses were intended to evaluate associations between clinical, structural, and operative variables rather than to establish causal relationships or develop a validated prediction model. Accordingly, the regression analyses should be interpreted as exploratory assessments of association within the study cohort. A *p*-value < 0.05 was considered statistically significant.

Missing data were assessed prior to analysis. Cases with incomplete key variables were excluded from multivariable models.

### 2.5. Ethical Considerations

The study protocol was reviewed and approved by the Institutional Ethics Committee of the Emergency County Hospital of Ploiești (approval no. 13741/24 March 2023). Due to the retrospective design and use of anonymized data, the requirement for informed consent was waived in accordance with institutional policies and applicable ethical standards.

## 3. Results

Baseline characteristics differed between groups (Table 1). Patients with recurrent hernia were older (42.1% vs. 34.2%, *p* = 0.012) and had higher rates of obesity (58.6% vs. 50.1%, *p* = 0.009), diabetes (28.3% vs. 20.2%, *p* = 0.004), and ASA III–IV status (45.2% vs. 32.0%, *p* < 0.001). Smoking rates were similar between groups (*p* = 0.58).

Hernia complexity differed between groups (Table 2). Recurrent hernias were more frequently associated with W3 defects (29.7% vs. 17.2%, *p* < 0.001) and lateral location (21.7% vs. 13.0%, *p* < 0.001). In contrast, W2 defects (46.6% vs. 57.5%, *p* = 0.002) and midline location (78.3% vs. 87.0%, *p* < 0.001) were more common in primary hernias.

Operative burden was higher in the recurrent hernia group (Table 3). Operative time was longer (152 ± 38 vs. 128 ± 30 min, *p* < 0.001), and length of hospital stay was increased (8.7 ± 3.5 vs. 7.1 ± 3.0 days, *p* < 0.001).

Surrogate markers of technical difficulty were significantly more frequent in recurrent hernia (Table 4). Operative time > 150 min (49.0% vs. 26.5%), W3 defect overlap (29.7% vs. 17.2%), and high-risk profile (62.4% vs. 41.3%) were all more common compared to primary hernia (all *p* < 0.001).

Hospital resource utilization was higher in the recurrent hernia group (Table 5). Prolonged hospitalization (>8 days) (54.8% vs. 35.2%, *p* < 0.001), overall complications (25.2% vs. 17.8%, *p* = 0.006), and reintervention rates (4.1% vs. 2.0%, *p* = 0.041) were all increased compared to primary hernia.

In multivariable logistic regression comparing recurrent and primary hernia cases, W3 defects (OR 2.41, 95% CI 1.75–3.31, *p* < 0.001), prolonged operative time (>150 min; OR 2.08, 95% CI 1.52–2.86, *p* < 0.001), ASA III–IV status (OR 1.76, 95% CI 1.28–2.41, *p* < 0.001), and obesity (BMI ≥30 kg/m^2^; OR 1.43, 95% CI 1.05–1.95, *p* = 0.021) were independently associated with recurrent hernia (Table 6). Age >65 years was not significantly associated with recurrence status (*p* = 0.19). These findings should be interpreted as characteristics associated with recurrent hernia cases rather than causal predictors of recurrence.

Composite operative burden differed between groups (Table 7). Low burden was more frequent in primary hernia (42.4% vs. 21.7%, *p* < 0.001), while high burden was more common in recurrent hernia (39.7% vs. 18.7%, *p* < 0.001). No difference was observed for moderate burden (*p* = 0.91).

## 4. Discussion

### 4.1. Principal Findings

This large cohort study demonstrates that recurrent incisional hernia is associated with increased patient vulnerability, greater structural complexity, longer operative time, prolonged hospitalization, and worse postoperative outcomes compared with primary hernia. Patients with recurrent hernia exhibited a more adverse baseline clinical profile, including higher rates of obesity, diabetes, advanced ASA status, as well as a higher prevalence of large (W3) defects and lateral localization. These factors are likely to have contributed to the longer operative time, prolonged hospitalization, and higher postoperative complication rates observed in this group. Nevertheless, recurrent hernia remained independently associated with postoperative complications after adjustment for the principal measured confounders in multivariable analysis. Because of the retrospective design and the absence of several potentially relevant variables, residual confounding cannot be excluded.

### 4.2. Recurrent Hernia as a Marker of Increased Surgical Complexity

Beyond descriptive findings, this study supports a conceptual shift in the understanding of recurrent incisional hernia. Rather than representing a simple failure of prior repair, recurrence may be viewed as a marker of increased structural complexity, technical difficulty, and patient-related vulnerability.

This interpretation is consistent with evidence showing that recurrent incisional hernia reflects not only mechanical failure but also impaired tissue remodeling and biological alterations that weaken abdominal wall integrity and promote recurrence [15,16,17,18,19,20,21,22,23,24,25,26].

The higher operative burden observed among patients with recurrent hernia should not be interpreted as being attributable solely to recurrence itself. Patients with recurrent hernia exhibited a substantially more adverse baseline clinical profile, including greater comorbidity burden and more complex defect characteristics, both of which are likely to contribute to longer operative time, prolonged hospitalization, and postoperative complications. Although multivariable adjustment accounted for the principal measured confounders, residual confounding cannot be excluded because of the retrospective design and limited availability of some clinically relevant variables.

Recurrent hernia is associated with greater operative difficulty because previous repairs often result in fibrosis, distorted anatomical planes, and prior mesh placement, creating a more challenging operative field. These technical challenges, together with the greater burden of comorbidities observed in recurrent cases, likely contribute to increased resource utilization and poorer postoperative outcomes [27,28].

Overall, recurrent incisional hernia should be regarded as a marker of progressive disease rather than simply a failed previous repair. This perspective may help explain its association with greater operative burden and worse postoperative outcomes observed in the present study.

### 4.3. Novelty in Relation to the Prior Literature

Previous studies have consistently shown that recurrent incisional hernia repair is associated with increased technical complexity, greater resource utilization, and less favorable clinical outcomes. However, these aspects have generally been evaluated separately through individual risk factors or isolated outcome measures. The principal contribution of the present study is a comprehensive comparison of primary and recurrent incisional hernias within a large homogeneous cohort undergoing elective retromuscular repair based on the Rives–Stoppa principle. Our findings demonstrate that recurrent hernias are associated with greater structural complexity, longer operative time, prolonged hospitalization, and higher postoperative complication rates. Furthermore, recurrent hernia remained independently associated with postoperative complications after adjustment for the principal measured confounders, supporting its role as a marker of increased surgical complexity rather than simply a history of previous repair. This concept is consistent with previous literature demonstrating that incisional hernia repair encompasses a broad spectrum of procedural complexity influenced by both patient-related factors and anatomical characteristics [1,29,30,31].

### 4.4. Clinical Implications

Our findings reinforce previous evidence identifying obesity, diabetes, and increased operative complexity as important determinants of postoperative complications following incisional hernia repair [22,32,33,34].

From a clinical perspective, recurrent incisional hernia should be recognized as a high-burden condition that may justify referral to specialized abdominal wall reconstruction centers with expertise in complex cases.

### 4.5. Strengths and Limitations

This study has several notable strengths. It includes a large and well-characterized cohort, allowing for a robust comparison between primary and recurrent incisional hernia. The integration of patient-related, structural, operative, and outcome variables provides a comprehensive assessment of the associations between recurrent hernia, surgical complexity, and postoperative outcomes.

However, several limitations should be acknowledged. First, outcome assessment was limited to in-hospital events because information regarding 30-day outcomes, long-term recurrence, chronic pain, mesh-related complications, surgical-site occurrences, hospital readmissions, and patient-reported outcomes was not consistently available.

Second, the retrospective single-center design introduces the potential for selection bias, limits causal inference, and may reduce the generalizability of the findings.

Third, postoperative complications were analyzed as a composite endpoint and were not graded according to a standardized severity classification such as the Clavien–Dindo system.

Fourth, residual confounding cannot be excluded because several technical and intraoperative variables, as well as detailed information regarding previous hernia repairs, were not consistently available.

Finally, the study was restricted to patients undergoing elective open retromuscular repair based on the Rives–Stoppa principle. Although this methodological homogeneity reduced treatment-related variability, the findings may not be directly applicable to minimally invasive approaches, emergency procedures, or institutions with different surgical expertise and perioperative management protocols. External validation in diverse clinical settings is warranted.

## 5. Conclusions

In this retrospective cohort of patients undergoing elective retromuscular incisional hernia repair, recurrent incisional hernia was associated with greater structural complexity, longer operative time, prolonged hospitalization, and worse postoperative outcomes compared with primary incisional hernia. Recurrent hernia remained independently associated with postoperative complications after adjustment for the principal measured confounders and may serve as a marker of increased surgical complexity. Further multicenter studies with long-term follow-up are warranted to validate these findings.

## Figures and Tables

**Table 1:** Baseline characteristics according to hernia status (*N* = 1262).

Variable	Primary Hernia (*n* = 972)	Recurrent Hernia (*n* = 290)	*p*-Value
**Age > 65 years**	332 (34.2)	122 (42.1)	0.012
**BMI ≥ 30 kg/m^2^**	487 (50.1)	170 (58.6)	0.009
**Diabetes mellitus**	196 (20.2)	82 (28.3)	0.004
**Smoking**	191 (19.6)	61 (21.0)	0.58
**ASA III–IV**	311 (32.0)	131 (45.2)	<0.001

Data are presented as *n* (%). *p*-values from the chi-square test.

**Table 2:** Hernia complexity and structural characteristics.

Variable	Primary Hernia	Recurrent Hernia	*p*-Value
**W3 defect (>10 cm)**	167 (17.2)	86 (29.7)	<0.001
**W2 defect (4–10 cm)**	559 (57.5)	135 (46.6)	0.002
**Midline location**	846 (87.0)	227 (78.3)	<0.001
**Lateral hernia**	126 (13.0)	63 (21.7)	<0.001

**Table 3:** Operative burden according to hernia status.

Variable	Primary Hernia	Recurrent Hernia	*p*-Value
**Operative time (min), mean ± SD**	128 ± 30	152 ± 38	<0.001
**Length of hospital stay (days)**	7.1 ± 3.0	8.7 ± 3.5	<0.001

**Table 4:** Surrogate markers of technical difficulty.

Variable	Primary Hernia	Recurrent Hernia	*p*-Value
**Operative time >150 min**	258 (26.5)	142 (49.0)	<0.001
**W3 defect + recurrence overlap**	167 (17.2)	86 (29.7)	<0.001
High-risk profile (≥2 factors) *	401 (41.3)	181 (62.4)	<0.001

* High-risk profile defined as ≥2 of the following: obesity, diabetes, ASA III–IV, W3 defect.

**Table 5:** Hospital resource utilization.

Variable	Primary Hernia	Recurrent Hernia	*p*-Value
**Prolonged hospitalization (>8 days)**	342 (35.2)	159 (54.8)	<0.001
**Any complication**	173 (17.8)	73 (25.2)	0.006
**Reintervention**	19 (2.0)	12 (4.1)	0.041

**Table 6:** Factors associated with recurrent versus primary hernia. Outcome: recurrent hernia (yes/no).

Variable	Odds Ratio (OR)	95% CI	*p*-Value
**W3 defect**	2.41	1.75–3.31	<0.001
**Operative time (>150 min)**	2.08	1.52–2.86	<0.001
**ASA III–IV**	1.76	1.28–2.41	<0.001
**BMI ≥ 30 kg/m^2^**	1.43	1.05–1.95	0.021
**Age > 65 years**	1.22	0.90–1.65	0.19

**Table 7:** Multivariable logistic regression for postoperative complications. Outcome: any complication (yes/no).

Variable	Odds Ratio (OR)	95% CI	*p*-Value
**Recurrent hernia**	1.54	1.10–2.15	0.012
**Operative time > 150 min**	1.88	1.35–2.62	<0.001
**ASA III–IV**	1.63	1.18–2.25	0.003
**BMI ≥ 30 kg/m^2^**	1.41	1.02–1.94	0.036
**W3 defect**	1.72	1.21–2.45	0.002

## Data Availability

The data presented in this study are available on request from the corresponding author. The data are not publicly available due to patient confidentiality.
